# Impact of Nutritional Epigenetics in Essential Hypertension: Targeting microRNAs in the Gut-Liver Axis

**DOI:** 10.1007/s11906-021-01142-9

**Published:** 2021-05-07

**Authors:** Rachel M. Golonka, Johnathan Kawika Cooper, Rochell Issa, Pratyush Pavan Devarasetty, Veda Gokula, Joshua Busken, Jasenka Zubcevic, Jennifer Hill, Matam Vijay-Kumar, Bindu Menon, Bina Joe

**Affiliations:** 1 Microbiome Consortium, Center for Hypertension and Precision Medicine, Department of Physiology and Pharmacology, The University of Toledo College of Medicine and Life Sciences, Block Health Science Bldg, 3000 Arlington Ave, Toledo, OH 43614 USA; 2 The University of Toledo College of Medicine and Life Sciences, Toledo, OH USA; 3 Department of Physiological Sciences and Center for Environmental and Human Toxicology, University of Florida Genetics Institute, Interdisciplinary Program in Biomedical Sciences Neuroscience, College of Veterinary Medicine, University of Florida, Gainesville, FL 32611 USA; 4 Department of Medical Education, University of Toledo College of Medicine and Life Sciences, Room 3105B, CCE Bldg, 2920 Arlington Ave, Toledo, OH 43614 USA

**Keywords:** Gut microbiome, Vasculature, Renin-angiotensin-aldosterone system, Hyperlipidemia, Bariatric surgery

## Abstract

**Purpose of Review:**

To review the current knowledge on interactions between dietary factors and microRNAs (miRNAs) in essential hypertension (EH) pathogenesis.

**Recent Findings:**

There exists an integration of maintenance signals generated by genetic, epigenetic, immune, and environmental (e.g., dietary) factors that work to sustain balance in the gut-liver axis. It is well established that an imbalance in this complex, intertwined system substantially increases the risk for EH. As such, pertinent research has been taken to decipher how each signal operates in isolation and together in EH progression. Recent literature indicates that both macro- and micronutrients interrupt regulatory miRNA expressions and thus, alter multiple cellular processes that contribute to EH and its comorbidities. We highlight how carbohydrates, lipids, proteins, salt, and potassium modify miRNA signatures during EH. The disruption in miRNA expression can negatively impact communication systems such as over activating the renin-angiotensin-aldosterone system, modulating the vascular smooth muscle cell phenotype, and promoting angiogenesis to favor EH. We also delineate the prognostic value of miRNAs in EH and discuss the pros and cons of surgical vs dietary prophylactic approaches in EH prevention.

**Summary:**

We propose that dietary-dependent perturbation of the miRNA profile is one mechanism within the gut-liver axis that dictates EH development.

## Introduction

**Fig. 1:**
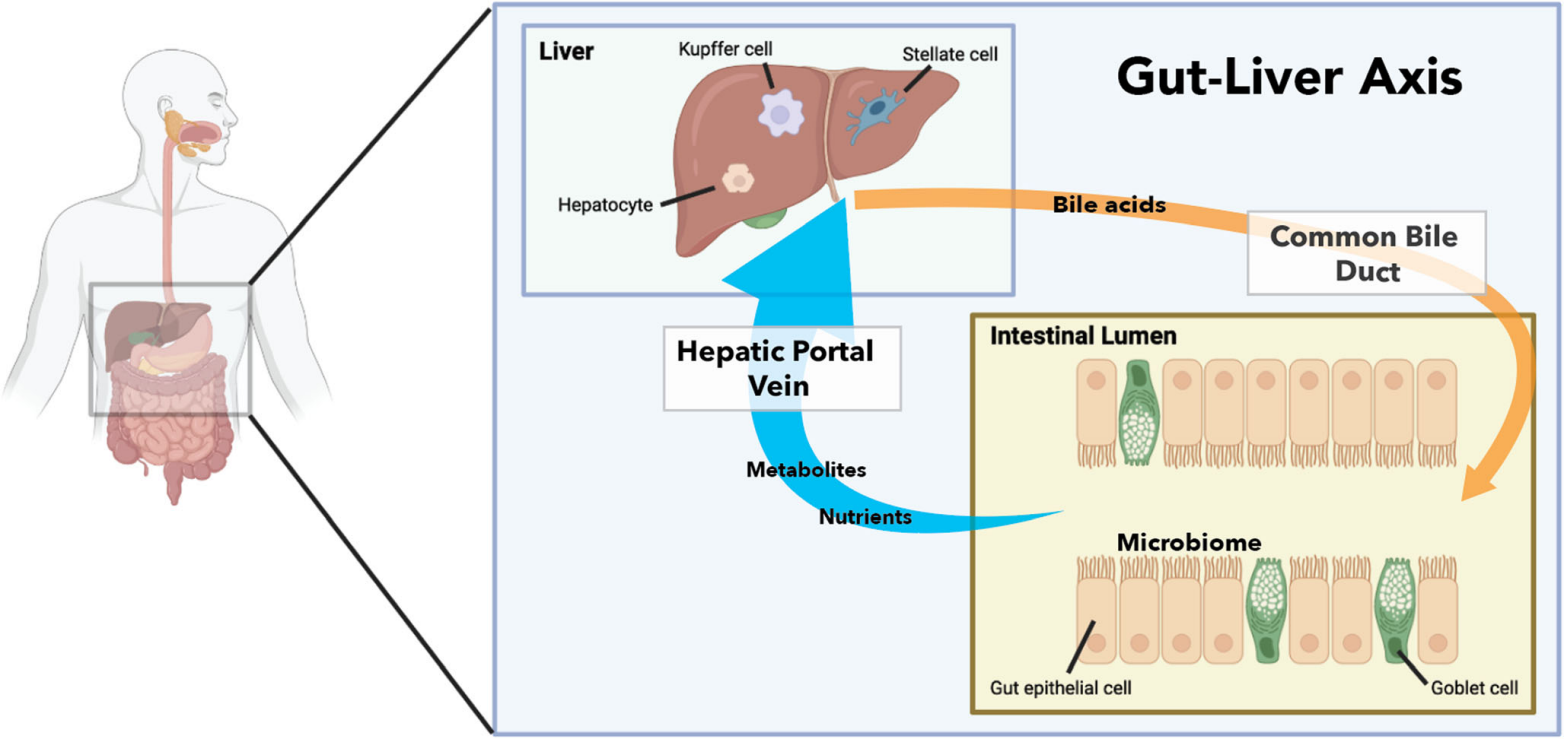
Bidirectional communication between the gut and the liver is required to maintain physiological homeostasis. The liver supplies bile, which is an aqueous solution of bile acids, bilirubin, organic solutes, and hormones, for nutrient assimilation, immune system stimulation, and intestinal development. The hepatic portal vein transfers venous blood enriched with nutrients and metabolites from the gut to the liver, which instigates xenobiotic metabolism and immune cell activation

An appropriate bidirectional crosstalk within the gut-liver axis (GLA) is essential to sustain physiological homeostasis. As summarized in Fig. 1, the liver initiates an enterohepatic relationship by synthesizing and metabolizing a variety of endogenous solid constituents, such as bile salts, bilirubin, phospholipids, and cholesterol [1]. These components are packaged with water as bile and deposited into the biliary tract for storage in the gallbladder. Upon ingestion of food, the gallbladder is signaled to contract and secrete bile into the small intestine for assimilation of nutrients. Food (e.g., indigestible carbohydrates like dietary fiber) that is not hydrolyzed by host digestive enzymes travels to the large intestine and is catabolized by commensal microorganisms known as the gut microbiota [2]. At the same time, host compounds like bile salts and bilirubin that enter the colon are susceptible to biotransformation into secondary-derived microbial products [3, 4]. This collection of dietary, host-derived, and microbial-derived components is transported via portal vein to the liver, where absorption of such contents can dictate the degree of immune stimulation and thus, inflammatory responses in the liver. For instance, gut-derived products can activate IL-6 production from Kupffer cells (resident macrophages in the liver), which have been suggested to stimulate acute phase protein production from hepatocytes [5]. Importantly, acute phase proteins thereafter impact the gut microbiota to complete the bidirectional communication circle, as we and others have shown that gut microbiota stimulation of lipocalin-2 limits the bioavailability of iron and therefore restricts the growth of iron-dependent pathobionts in the intestine [6–8].

In cases of gut barrier dysfunction, more commonly termed a “leaky gut,” excess release of microbial components can serve as ligands for pattern recognition receptors that instigate excessive inflammation and increase the risk for hepatic tissue damage [9]. One of the major responses upon liver injury is the transactivation of hepatic stellate cells into pro-fibrotic myofibroblasts [10, 11]. End-stage fibrosis (i.e., cirrhosis) can obstruct portal vein blood flow, resulting in portal hypertension characterized by intrahepatic vascular resistance and elevated blood pressure [12]. Alongside portal hypertension, our group was one of the first to reveal the link between gut microbiota and salt-sensitive hypertension [13••], where our later studies identify more mechanistic insights to how disruption in the GLA negatively impacts blood pressure [14–16, 17•]. A recent review by Simbrunner et al. elegantly introduces several molecular mechanisms for GLA signaling in portal hypertension, including host-microbiome co-metabolism [18]. Another respective review by Guo et al. describes the therapeutic potential of microRNAs (miRNAs) in regulating hepatic stellate cell differentiation to treat portal hypertension [19••]. Furthermore, a meta-analysis by Marques et al. characterizes miRNA signatures in the major blood pressure regulatory organs from rodent models and human studies of essential hypertension [20••].

**Fig. 2:**
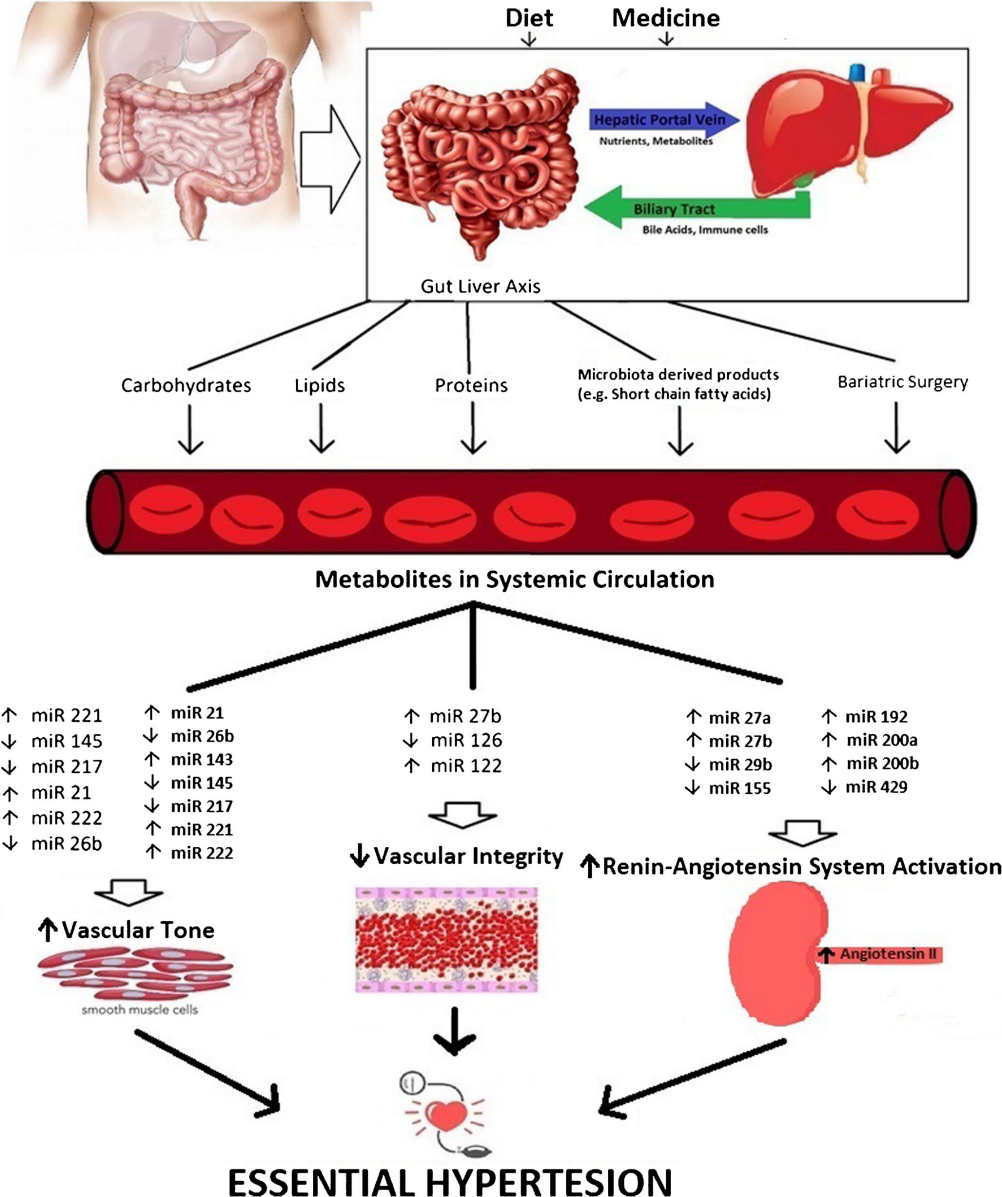
Altered miRNA expressions within the gut-liver axis from response to diet promote essential hypertension. Dietary components such as carbohydrates, lipids, and proteins can alter the miRNA signatures that favor for increased vascular tone and renin-angiotensin-aldosterone system activation, but reduced vascular integrity, which all collectively contribute to essential hypertension development. In addition, therapeutic approaches such as probiotics to increase short chain fatty acid levels and/or bariatric surgery can affect miRNA-dependent regulation of vascular function and thus, increase risk for essential hypertension

Herein, we expand by compiling available evidence on how dietary perturbation in the GLA alters miRNA expressions during essential hypertension (as summarized in Fig. 2). This includes examining the reported effects of macro- and micronutrients, such as carbohydrates, lipids, proteins, salt, and potassium. We also discuss the prognostic and therapeutic value of miRNAs in essential hypertension and outline potential dietary factors that could prove fruitful for prevention and management of essential hypertension via modulation of miRNAs.

## microRNAs in the Gut-Liver Axis

miRNAs are single-stranded, non-coding RNAs approximately 21–25 nucleotides long that are required for nearly all cellular processes related to animal and plant development [21]. Processing from immature to mature miRNAs is a two-step system: (i) primary miRNA transcripts are cleaved by the RNase III nuclear enzyme Drosha and (ii) the released stem-loop pre-miRNA is cleaved by the RNase III cytosolic enzyme Dicer to make a mature miRNA [22, 23]. The mature miRNA then forms an effector RNA-induced silencing complex in collaboration with members of the Argonaute family of proteins to repress protein-coding messenger RNAs via degradation [24]. When considering that miRNAs are predicted to regulate around 30% of protein-encoding genes [25], it is not surprising that miRNAs have been implicated in a variety of pathophysiological outcomes. It is noteworthy that the miRNA signature is specifically altered in liver diseases and can dictate either pro- or anti-inflammatory, pro- or anti-fibrotic, and oncogenic- or tumor-suppressive gene expressions [26]. Interestingly, miRNAs and the gut microbiota have a reciprocal regulatory interaction on each other in both physiological and pathological conditions [27]. This suggests that miRNAs may be an important GLA component in modulating both liver and gut homeostasis.

In portal hypertension, most research to date has focused on miRNA signatures associated with hepatic cirrhosis and splenomegaly as the causation and secondary consequence, respectively. The impact of miRNAs on hepatic stellate cells and other signaling pathways in cirrhosis has been reviewed in-depth [28–31], whereas the molecular role of miRNAs in hypersplenism has only been recently described. Whole-genome microarray analysis has identified a distinct miRNAome in the enlarged spleens of animals with partial portal vein ligation-induced hypertension, including twenty-two downregulated miRNAs that would normally suppress fibrotic related mRNAs (e.g., *Col1a1*, *Serpine1*) [32•]. Intriguingly, miRNA-615-3p was found to be highly expressed in the splenic macrophages of cirrhosis-related portal hypertensive patients who underwent splenectomy [33]. Further analysis revealed that excess miRNA-615-3p repressed the ligand-dependent nuclear receptor corepressor, followed by enhanced PPARγ-dependent phagocytic capacity from macrophages [34], which delineates one potential cellular mechanism of hypersplenism during portal hypertension. Considering the reported impacts of the miRNA-mRNA network on physiology, we propose that miRNAs may also be responsible for the GLA-dependent mechanisms in essential hypertension (EH).

## Prognostic Value of microRNAs in Essential Hypertension

EH is a complex, multi-factorial, polygenic condition with heterogeneous etiological risk factors. Extensive molecular genetic research has identified single nucleotide polymorphisms in several genes for Mendelian categorized EH [35]. High-salt intake, excessive alcohol, stress, and low potassium consumption have also been pinpointed as dominant environmental contributors to EH pathogenesis [36]. Most recently, epigenetics has emerged as a novel and powerful hallmark of EH progression, which encompasses DNA methylation, post-translational histone modifications, and miRNAs [37, 38]. By studying epigenetics, the heredity aspects of EH and its phenotypes may be clarified [39, 40]. In this scenario, the miRNAs are clinically relevant due to their capability to affect several gene expressions.

Importantly, specific miRNAs have been suggested as potential stable circulating biomarkers for EH diagnostic applications. This includes a collection of miRNAs reported to be either upregulated (e.g., miR-1, miR-21, miR-122, miR-198, miR-202-3p, miR-208b, miR-499, miR-505, miR-510, miR-575, miR-1183) [41••, 42, 43•, 44–50] or downregulated (e.g., miR-9, miR-10a-5p, miR-26b, miR-29a, miR-29b, miR-29c, miR-30e-5p, miR-126, miR-133a, miR-136, miR-143, miR-144-3p, miR-145, miR-146a) [43•, 48, 50–55] in circulation of EH patients compared to healthy controls. This miRNA profile is correlated with sub-clinical cardiovascular diseases such as left ventricular hypertrophy, cardiac remodeling, carotid intima-media thickness, nephropathy, albuminuria, endothelial dysfunction, and vascular dysfunction [42, 44–46, 48, 51, 52, 54, 56–58]. In addition, miRNAs are associated with alterations in specific cellular communication systems like renin-angiotensin-aldosterone system, vascular smooth muscle modulation, angiogenesis, and mineral ion binding, which all reportedly contribute to EH pathogenesis [43•, 44, 49–51]. Investigation of EH in rodent and zebrafish models identified miR-27a and miR-27b as additional pro-hypertensive miRNA candidates, as their increased presence in extracellular vesicles was linked to reduction in endothelial nitric oxide synthase phosphorylation, impaired angiotensin-(1-7)-dependent vasodilation, and increased angiogenesis [59••, 60]. Overall, analyses of the circulating miRNA signatures present a prognostic tool as well as pave the way for precision medicine of EH patients.

## Nutritional Impact on microRNA Signatures in Essential Hypertension

There exists multiple environmental and genetic factors that can perturb the gut microbiota to cause dysbiosis and aid in the blooming of opportunistic pathogenic bacteria at the expense of beneficial commensals [61]. Recent studies highlight a strong association between gut dysbiosis and EH [62] and suggest that the microbiome composition contributes to EH pathogenesis [63••]. Thus, interventions at the gut microbiota level to normalize blood pressure and vascular function may be beneficial. Guidelines from the American College of Cardiology and American Heart Association suggest dietary lifestyle changes to manage and prevent EH [64••]. In addition, the emerging field of nutritional epigenetics [65] show that dietary perturbations within the GLA may have downstream effects on miRNA signatures. In this section, we discuss the potential of leveraging nutritional epigenetics for EH treatment.

### Carbohydrates

It is well recognized that a Western-style diet, composed of excessive simple carbohydrates and saturated fats, is a risk factor for EH development [66]. One ingredient that has received heavy research attention to date is high-fructose corn syrup found in sweetened beverages and Westernized foods. Consumption of dietary fructose alone is sufficient to elevate blood pressure in adolescents and adults [67–69] whereas glucose ingestion does not change blood pressure [69]. As such, fructose has been acknowledged as an independent risk factor for EH progression for humans [70] and rodent hypertensive models [71, 72]. Several studies delineate the role of fructose on sodium and electrolyte balance, nitric oxide (NO) bioavailability, oxidative stress, and vascular integrity [73, 74], which in turn promotes EH.

In view of the evidence, it is plausible that fructose could alter miRNA expression in the GLA during EH. A study by Sud et al. found that a high-fructose diet significantly altered the expression levels of certain miRNAs related to lipid metabolism [75••]. Intriguingly, some of the same miRNAs are associated with regulating endothelial function and blood pressure. For instance, expression levels of miR-19b and miR-101a were suppressed following a high-fructose diet [75••], and this could attribute to EH pathogenesis considering that both miRNAs demonstrate anti-atherogenic properties [76]. While this evidence suggests miR-101a as anti-hypertensive, this miRNA is reportedly a part of an underlying mechanism of increased diastolic blood pressure from air pollution exposure [77], highlighting the complexity of miRNA function. Overconsumption of fructose also upregulated the expression of miR-145a [75••], a candidate pro-hypertensive miRNA and potential biomarker for diagnosing EH as silencing miR-145a in spontaneously hypertensive rats protected against EH by restoring NO metabolism [78]. These studies collectively suggest that future research should investigate the potential role of fructose-induced augmentation of miR-145a in EH.

In addition to the reported effects of fructose on miRNAs, other evidence suggests the renin-angiotensin-aldosterone system (RAAS) as an intermediate in fructose-miRNA interaction. As low blood pressure initiates the RAAS cascade to elevate blood volume and arterial tone, the liver is the primary site of angiotensinogen production, a precursor for angiotensin II (Ang II), which acts as a potent vasoconstrictor and promotes sodium and water reabsorption via angiotensin II type I receptor (AT1) in the kidney, adrenal cortex, arterioles, and brain [79]. Interestingly, excessive levels of intra-renal Ang II [80] and increased AT1 signaling [81] have been implicated in the hypertensive effects from high a fructose diet. Moreover, miR-155 functions downstream of Ang II signaling as a negative feedback regulator, a suggested protective mechanism against cardiac hypertrophy [82••]. This posits that the negative regulation of Ang II by miR-155 might be impaired during EH; however, further studies are required to delineate this possibility.

The role of miRNAs in fructose-induced EH may also be mediated via advanced glycation end (AGE) products. Fructose, like other reducing sugars, can nonenzymatically react with free amino groups from proteins, lipids, or nucleic acids through the Maillard reaction (glycation) to produce AGE [83, 84]. It is important to note that glucose is the lesser/slower reactive sugar that participates in glycation when compared to fructose [84]. Associative studies have found that AGE are significantly higher in EH patients [85], as they reportedly contribute to endothelial dysfunction [86]*.* A study by Wu et al. demonstrates that AGE can suppress miR-200b and miR-200c, leading to unregulated RhoA/ROCK2 signaling during endothelial injury [87]; however, whether this relates to EH etiology remains unknown. Thus, the role of elevated AGE [88] and/or impaired endothelial mechanotransduction [89] as a result of dietary sugar consumption needs further investigation in EH. Noteworthy is that chronic ingestion of sucrose reportedly increased circulatory miR-21 and miR-223 [90]. Therefore, this miRNA change may be a compensatory response when considering that (i) EH patients have elevated levels of miR-21 in circulation, (ii) delivery of miR-21 lowered blood pressure in spontaneously hypertensive rats via restoration of mitochondrial function, and (iii) miR-223 is anti-atherogenic by targeting β1 integrin [91•, 92]. Overall, future studies should directly explore whether perturbation of the GLA by fructose and/or sucrose changes the miRNA profile to favor EH.

### Lipids and Fatty Acids

Adoption of a sedentary lifestyle and dietary changes (i.e., excessive intake of lipids) that lead to a positive energy balance can cause hyperlipidemia. In addition to lipid overconsumption, which leads to hyperlipidemia, as a risk factor for EH progression [93], there exists is a coupling effect for dietary fats to aggravate developed EH [94]. Impressively, mothers consuming high-fat diet (HFD) during the perinatal period increases the probability for offspring to exhibit fetal reprogramming toward EH development [95]. Alterations in the gut microbiota and their metabolite profile [95] have been suggested to contribute to the intergenerational transfer of EH risk, and this may reflect the changes in miRNA composition. For one, prolonged maternal exposure to HFD was associated with downregulation of miRNA processing in the offspring, which contributed to fetal cardiac hypertrophy [96••]. Additionally, a recent study by Mantilla-Escalante et al. finds that postprandial lipemia causes specific miRNA responses including tissue enrichment of miR-206-3p, miR-543-3p, miR-466c-5p, miR-27b-5p, miR-409-3p, miR-340-3p, miR-1941-3p, miR-10a-3p, miR-125a-3p, and miR-468-3p [97•]. Analyses of circulating miRNA profiles in EH associated with hyperlipidemia conditions show an increase for miR-21, -146a, -221, -143, -34a, and miR-204 in plasma levels, elevated miR-126, -146a, -223, -222, and miR-214 and reduced miR-143, miR-10a, and miR-145 in platelets, and increased miR-222, -221, -210, and miR-34a and decreased miR-223, -214, -146a, -143, -10a, and miR-145 in platelet derived vesicles [98]. Thus, it could be theorized that the vascular hyperreactivity and cardiac remodeling during EH is due to hyperlipidemia-dependent changes in miRNA expressions, such as downregulation of miR-10a, miR-139b, miR-206, and miR-222 and/or upregulation of hsa-miR-223-3p, hsa-miR-21-5p, and hsa-miR-146a-5p [99, 100••]. Furthermore, miR-21 is upregulated in enriched lipid environments and disrupts the remodeling of vascular smooth muscle cells during EH [50]*.*

There are four types of fatty acids: saturated, monosaturated, polyunsaturated, and trans-fat. Comparatively, saturated, monounsaturated, and trans-fats, but not polyunsaturated fatty acids (e.g., ω3 and ω6), are associated to EH [101]. In line with this, HFD-fed rats that consumed linoleic acid (ω6) were found to have lower miR-27a (pro-hypertensive candidate) and restoration of miR-143 (normally blunt in EH) [102], suggesting that polyunsaturated fatty acids could be therapeutic against hyperlipidemia-associated EH. Accordingly, much research has focused on understanding the role of saturated fatty acids in EH progression because it is the most consumed form of dietary lipids. Palmitic acid, in particular, has received much attention for obesity-associated EH by upregulating endothelin-1 levels through induction of endoplasmic reticulum stress [103]. Multiple mechanisms may exist via which saturated fatty acids influence EH, including damaging the integrity of the inner lining of blood vessels in the vascular endothelium, diminishing NO production, increasing oxidative stress, exacerbating inflammation, and promoting the activation of RAAS [104]. It is noteworthy that postprandial lipemia resulting from ingestion of enriched saturated fatty acids—mostly composed of palmitic acid—downregulated miR-300 and miR-369-3p but upregulated miR-495-3p, miR-129-5p, and miR-7-2-3p in peripheral blood mononuclear cells [105]. This connection among lipemia, miRNA, and EH is evident but requires additional mechanistic studies to confirm how these changes in miRNA levels impact EH etiology and progression.

### Proteins and Amino Acids

Dietary Approaches to Stop Hypertension (DASH) studies [106] indicate that long-term intake of a high-protein diet, at the expense of carbohydrates, from either animal or plant sources has protective benefits to lower the risk of EH [107] and to maintain reduced blood pressure even after weight loss [108]. While no studies have currently looked at the direct effects of protein on miRNA expression in EH, evidence indicates that certain amino acid intermediates may play a role in EH pathology. For instance, homocysteine and asymmetrical dimethylarginine (ADMA) are two byproducts from post-translational modification (i.e., methylation) of arginine [109]. Metabolism of methionine is another source for homocysteine [110]. Hyperhomocysteinemia (serum homocysteine levels >10 μmol/L) [111, 112] and significantly increased ADMA levels [113] have become more widely recognized as risk factors for EH development. ADMA is primarily known to inhibit NO bioavailability and induce endothelial dysfunction [113], whereas homocysteine can also inhibit NO synthesis while promoting oxidative stress. Recently, Li et al. found that homocysteine can competitively inhibit Ang II when activating AT1 in the RAAS pathway [114••]. Homocysteine is also well known for stimulating proliferation of vascular smooth muscle cells [115], reportedly via miR-143 hypermethylation [116] and blunted miR-145/CD40 [117, 118] but this can be abated by (i) miR-217 suppression of the N-methyl-D-aspartic acid receptor [119], (ii) miR-217 promotion of senescence [120], or (iii) miR-145 repression of PI3K/Akt/mTOR signaling [121••]*.* Furthermore, cardiac remodeling from homocysteine is linked to the differential expression of 11 miRNAs with miR-188 showing dramatic downregulation in HHcy cardiomyocytes [122]. Despite the reports that homocysteine is related to the phenotypical switches found in EH and that these changes are associated with specific miRNA signatures, these observations need to be confirmed in EH rodent models.

### Micronutrients

There are five main micronutrients that are known to regulate blood pressure: sodium, chloride, calcium, potassium, and magnesium [123]. Accordingly, the molecular compound sodium chloride has been heavily investigated in EH pathogenesis [124]. Mechanistically, high salt intake has been shown as pathological in EH by regulating immune responses [125], causing renal dysfunction [126], and modulating the gut microbiota and metabolic profile [127]. This also includes dysregulated sodium and water reabsorption, higher glomerular filtration rate, and increase of protein catabolism in EH patients [128]. In line with this, mice deficient in the sodium chloride cotransporter have increased blood pressure when fed a diet with high salt and low potassium [129].

High-throughput miRNA sequencing technology has identified 9 miRNAs suitable as biomarkers for salt-sensitive EH in humans, including upregulation of hsa-miR-15b-5p, hsa-miR-362-5p, and hsa-miR-361-5p, but downregulation of hsa-miR-19a-3p, hsa-miR-210-3p, hsa-miR-26b-3p, hsa-miR-382-5p, and hsa-miR-423-5p [130]. miRNA libraries have also been created for Dahl salt-sensitive and Lewis rats administered with either normal or high-salt diets, as Naraba and Iwai confirmed 91 previously reported miRNAs and uncovered 12 new miRNAs expressed in the kidney [131]. Interestingly, miR-429 is reportedly necessary for HIF-1α-mediated sodium excretion in response to high salt intake, whereas deficiency in this miRNA aggravated salt-sensitive EH [132]. In line with this, a recent study by Lu et al. finds that the circular RNA, termed circNr1h4, regulates fatty acid reductase 1 by sponging miR-155-5p, which contributes to renal injury during deoxycorticosterone acetate-salt hypertension [133]. Comparatively, miR-29b is indicated as potentially beneficial against renal fibrosis in salt-induced EH as it suppresses a wide array of genes that encode collagen [134]. Of note is that administration of a first generation β1-selective blocker (i.e., nebivolol, atenolol) substantially alleviated cardiac remodeling, hypertrophy, and fibrosis in salt-sensitive EH by attenuating miR-27a and miR-29a [135].

## Surgical vs Dietary Prophylactic Approaches to Prevent Essential Hypertension

### Bariatric Surgery

In association with hyperlipidemia, obesity is a prominent risk factor for EH [136]. Therapeutic approaches have included the coupling of a calorie deficit diet and intense exercise, but recent evidence suggests that surgical procedures such as bariatric bypass, adjustable gastric banding, vertical banded gastroplasty, and biliopancreatic diversion are more successful in maintenance of long-term weight loss and as such, lower the incidence of EH [137]. Sleeve gastrectomy and Roux-en-Y gastric bypass are the two most common types of bariatric surgeries that involve a partial resection of the stomach, which alters bile flow and metabolic pathways to stimulate weight loss in patients with a body mass index greater than 40 [138]. Interestingly, changes in the systemic profile of regulatory miRNAs have been noted by a marked decrease of circulating miR-140-5p, miR-122, miR-193a-5p, and miR-16-1 but an increase of miR-221 and miR-199a-3p following surgery-induced weight loss [139]. Urinary levels of miR-192, miR-200a, and miR-200b were also found to be upregulated following bariatric surgery [140]. Additionally, liver-specific miR-122, miR-885-5-p, and miR-192 were reduced to levels found in non-obese patients 3 months post-surgery [141]. Changes in the miRNA profile also corresponded with the suppression of pro-inflammatory genes in adipose tissue [142], which could attribute to how miRNAs switch toward an anti-inflammatory metabolic state after gastric bypass.

A recent meta-analysis further affirms that bariatric surgery significantly alters miRNA expressions: (i) downregulated hsa-miR-93-5p, hsa-miR-106b-5p, hsa-let-7b-5p, hsa-let-7i-5p, hsa-miR-16-5p, hsa-miR-19b-3p, hsa-miR-92a-3p, hsa-miR-222-3p, hsa-miR-142-3p, hsa-miR-140-5p, hsa-miR-155-5p, and rno-miR-320-3p, but (ii) upregulated hsa-miR-7-5p and hsa-miR-320c [143•]. Since miRNAs were positively correlated with reduced body mass index, percentage fat mass, blood glucose levels, and liver transaminases, these results have instigated the miRNAome as a potential diagnostic tool to indicate the success of bariatric surgery [144]. Considering the drastic weight loss experienced by patients after bariatric surgery, the high rates of EH remission 1 year after surgery are surprising [145]. However, a recent longitudinal genome-wide methylation study revealed that Roux-en-Y gastric bypass patients obtain novel CpG sites associated with dysregulation of systolic blood pressure, which has provided a plausible epigenetic mechanism to EH post-surgical treatment [146]. Future studies are necessary to delineate if the above changes in the miRNAome could be also contributing to the EH remission observed after bariatric surgery.

### Probiotics and Prebiotics: Targeting the Gut Microbiota

Novel dietary supplements are currently explored to implement either in the pre-hypertensive stage or as co-adjuvants with standard treatment plans for EH. When determining appropriate dietary therapeutics, it is important to note which metabolites are generated once the food is digested by the gut microbiota. In the proximal colon, for instance, saccharolytic (i.e., carbohydrate) fermentation by microbes can generate beneficial short chain fatty acids (SCFA) and vitamins B and K [147, 148]. Comparatively, proteolytic (i.e., amino acid) fermentation in the distal colon can generate branch-chain fatty acids but also some potentially detrimental metabolites such as ammonia and phenols [147, 148]. As such, one of the major mainstream options in EH management is the incorporation of either probiotics or prebiotics [149–152, 153•] into the diet to boost saccharolytic fermentation from beneficial commensal microbes and thus generate SCFA like acetate, butyrate, and propionate. This rationale is further supported by the clinical observations that EH patients have greater excretion and less absorption of SCFA [154••, 155, 156].

Dietary supplementation with probiotics may include commensal strains from *Lactobacillus* and *Bifidobacteria* whereas prebiotics encompass ingestion of dietary fibers as a nutritional source for the resident gut commensals. The blood pressure regulatory role of SCFA has been delineated in-depth with back-to-back reviews from Dr. Jennifer Pluznick [157, 158]. To summarize, administration of one type of SCFA or as a cocktail mixture has been found to reduce blood pressure by (i) activating olfactory G-protein coupled receptors in the kidney [159, 160] and (ii) acting as a histone deacetylase inhibitor [161]. Even though SCFA possess well-established epigenetic effects via inhibiting histone deacetylase function, only one article to date makes a connection between SCFA and miRNA in EH, as Weber et al. note that the miRNA-dependent hypertensive phenotype may be due to its regulation of SCFA receptors in the kidney, which can be normalized by hydrogen sulfide administration [162]. Future studies should aim to further understand the SCFA-miRNA-EH axis.

## Conclusion and Future Direction

This review highlights our current understanding of how miRNAs may influence EH progression in the context of the GLA. Specifically, we delved into the interplay between dietary factors and gut microbiota metabolites in the “turning on” vs “turning off” expression of certain miRNAs, which may dictate a pro-hypertensive vs normotensive state. Noting that our review is one of the first to make the connection among dietary factors, GLA-derived metabolites, and miRNAs in EH, additional research is needed to validate these correlations. The diagnostic value of miRNA in EH has been substantiated, but mechanistic studies are needed to identify the targeted miRNA for precision medicine in EH. Intriguingly, the study by Teng et al. demonstrates that miRNAs from exosome-like nanoparticles in ginger can positively affect the composition of gut microbiome and its metabolites [163], but whether this could be translated to alleviate EH has not yet been explored. Our review indicates that studying the differences between a Westernized style diet vs plant-based diet could provide prevention and/or treatment of EH. Other research also indicates that a Mediterranean diet may provide a beneficial alteration of the miRNA signatures and lower endothelial dysfunction [164]. We acknowledge that miRNAs may be one of many epigenetic factors contributing to EH and further research should determine if other non-regulatory RNAs, such as small and long regulatory RNAs or cyclic RNAs, may have similar implications as miRNAs in EH pathogenesis.

## Abbreviations

ω3: omega-3
ω6: omega-6
ADMA: asymmetrical dimethylarginine
AGE: advanced end product
AKT: protein kinase B
Ang I: angiotensin I
Ang II: angiotensin II
AT1: angiotensin II type I receptor
DASH: Dietary Approaches to Stop Hypertension
EH: essential hypertension
GLA: gut-liver axis
HFD: high-fat diet
HIF-1α: hypoxia inducible factor 1α
IL-6: interleukin-6
miRNA: microRNA
mTOR: mechanistic target of rapamycin
NO: nitric oxide
PI3K: phosphatidylinositol 3-kinases
PPARγ: peroxisome proliferator-activated receptor gamma
RAAS: renin-angiotensin-aldosterone system
ROCK2: Rho-associated protein kinase 2
RHOA: Ras homolog gene family, member A
SCFA: short-chain fatty acid
